# Relationship between longitudinal changes in type‐2 inflammation, immunoglobulin E sensitization, and clinical outcomes in young asthmatics

**DOI:** 10.1002/clt2.12066

**Published:** 2021-09-27

**Authors:** Nikolaos Tsolakis, Tiago Jacinto, Christer Janson, Magnus Borres, Andrei Malinovschi, Kjell Alving

**Affiliations:** ^1^ Department of Women's and Children's Health Uppsala University Uppsala Sweden; ^2^ University of Porto Porto Portugal; ^3^ Medical Sciences Uppsala University Uppsala Sweden; ^4^ ImmunoDiagnostics Thermo Fisher Scientific Uppsala Sweden

**Keywords:** asthma, NO, eosinophils, IgE

## Abstract

**Background:**

Asthma is a heterogeneous condition where biomarkers may be of considerable advantage in diagnosis and therapy monitoring. However, the changes in asthma biomarkers and immunoglobulin E (IgE) over the course of life has not been extensively investigated.

**Objective:**

To study longitudinal changes in type‐2 inflammatory biomarkers, IgE, and clinical outcomes, and the association between these changes, in young asthmatics.

**Methods:**

Asthmatics (age 10–35 years, *n* = 253) were examined at baseline and at a follow‐up visit, 43 [23–65] (median [range]) months later. Subjects were analyzed using the multi‐allergen tests Phadiatop and fx5 (ImmunoCAP) and grouped based on the baseline allergen‐specific IgE antibody (sIgE) concentration: <0.10, 0.10–0.34, and ≥0.35 kU_A_/L. The relationship between changes (Δ values) in type‐2 biomarkers (individualized fraction of exhaled nitric oxide [FeNO%], blood eosinophil [B‐Eos] count, total IgE [tIgE] and sIgE, lung function [% predicted forced expiratory volume in 1 second (FEV_1_) and FEV_1_/forced vital capacity (FVC)], and Asthma Control Test [ACT]) score were determined.

**Results:**

At follow up, FEV_1_ and FEV_1_/FVC had decreased (93.6% vs. 95.8%, and 93.4% vs. 94.7% of predicted, respectively [*p* < 0.001 both]), whereas ACT score had increased (21.6 vs. 20.6, *p* = 0.001). A significant decline in lung function was seen in subjects with sIgE ≥ 0.10 kUA/L, but not in those with undetectable sIgE (<0.10 kU_A_/L). Furthermore, tIgE and sIgE declined over time (*p* < 0.001 all) whereas FeNO% and B‐Eos count were not significantly changed. In univariate analysis, significant negative correlations between ∆B‐Eos count and ∆FeNO%, on one hand, and changes in lung function, on the other hand, were seen, and multivariate analysis showed an independent relationship between ΔFeNO%, and ΔFEV_1_ (*p* < 0.05) and ΔFEV_1_/FVC% (*p* < 0.01). Sex‐specific analysis showed that the independent association between ΔFeNO%, and ΔFEV_1_ remained only in females (*p* = 0.005), and there was a significant interaction with sex (*p* = 0.02).

**Conclusion:**

In young asthmatics, IgE levels declined over 43 months, whereas FeNO and B‐Eos remained unchanged. In spite of improved asthma control, an accelerated lung function decline was seen in patients with detectable sIgE at baseline, and the decline correlated with changes in type‐2 biomarkers. Particularly, the increase in individualized FeNO associated independently with decline in FEV_1_ in females.

## 1 INTRODUCTION

Asthma is a complex syndrome where clinical and biomarker‐assisted phenotyping can be useful in the development of novel therapies.^1^ However, it is uncertain how these phenotypic asthma characteristics vary over time. For example, it is well‐known that elevated fraction of exhaled nitric oxide (FeNO), a local biomarker of type‐2 inflammation, predicts asthma worsening in patients with allergic asthma, and it has been shown that changes in FeNO might be meaningful for the longitudinal assessment of asthma control.^2^, ^3^ In contrast, there is limited knowledge about the association between changes in blood eosinophil (B‐Eos) count, a systemic biomarker of type‐2 inflammation, and asthma outcome over time. However, a higher B‐Eos count is a risk factor for future exacerbations and poor asthma control.^4^, ^5^

Similar studies have focused on changes in lung function over time, in both children and adults with asthma, aiming to identify contributing factors for the outcome of asthma onset.^6^ In the relationship between lung function and asthma, total immunoglobulin E (tIgE) and atopy may have a central role, at least at younger ages. In children, when the lungs are growing and the risk for allergic sensitization is high, a high tIgE has been shown to be a negative factor for lung function development, whereas the impact of tIgE on lung function in middle‐aged individuals seems to be much weaker.^7^ Furthermore, Turner and coworkers have linked the presence of early onset atopy at one month of age to reduced lung function at 18 years of age.^8^

The phenotyping of asthma is still under debate, and many questions regarding the factors that determine the outcome of asthma remain unanswered. Based on the Minimally Invasive Diagnostic procedures in allergy, Asthma, or food hypersensitivity Study (MIDAS) cohort, we have profiled clinical outcomes and inflammatory biomarkers in relation to allergen‐specific IgE antibody levels (sIgE) in young asthmatics.^9^ In the present study, the participants were followed up after a median of 43 months (range 23–65) and the changes in asthma biomarkers over time was determined in order to identify different asthma phenotypes, and to examine associations with changes in clinical outcomes.

## 2 MATERIALS AND METHODS

### 2.1 Subjects

The MIDAS asthma cohort was recruited from primary and specialist care facilities in Uppsala, Sweden.^10^, ^11^ The original cohort comprised 408 young subjects with asthma and 118 random healthy control subjects, aged 10–35 years at baseline. A total of 341 subjects (253 with asthma) were re‐examined at a follow‐up 43 [23–65] months (median [range]) later. The inclusion criteria were physician‐diagnosed asthma and daily treatment with an inhaled corticosteroid (ICS) and/or an oral leukotriene‐receptor antagonist (LTRA) during at least 3 of the past 12 months. The present study includes the 253 asthmatics that participated in both examinations. The measurements were basically performed all around the year with the exception of vacation periods (Christmas holidays and the summer period from the end of June to middle of August).

### 2.2 Blood measurements

IgE antibodies were analyzed using two different multiallergen tests, one with a mix of nine aeroallergens (birch, timothy grass, mugwort, *Dermatophagoides pteronyssinus*, *Dermatophagoides farinae*, *Cladosporium herbarum*, cat, dog and horse; Phadiatop), and one with a mix of six food allergens (egg white, cod fish, cow's milk, soybean, wheat and peanut; fx5).^12^ Both sIgE and tIgE were measured in the ImmunoCAP system (Immunodiagnostics, Thermo Fisher Scientific). B‐Eos counts were analyzed at the Department of Clinical Chemistry and Pharmacology at Uppsala University Hospital using an automated cell counter (Cell‐Dyn Sapphire, Abbott).

### 2.3 Asthma symptoms and medication

Participants responded to questions regarding their asthma symptoms in the last 12 months.^13^ The degree of asthma control was assessed using the Asthma Control Test (ACT).^14^ The total ACT score ranges between 5 and 25, with a lower score pointing towards poorer asthma control. A score ≥20 reflects well‐controlled asthma. The mini Asthma‐Related Quality of Life Questionnaire (mAQLQ) consists of 15 questions. The score ranges from 1 to 7, with a lower score indicating poorer quality of life.^15^ Asthma attacks were self‐reported, requiring at least a doubling of ICS, and subjects who were informed about the treatment of an asthma attack at home, were divided into those having had a recent (last 3 months) asthma attack and those who had not. The use of ICS and LTRA was recorded in the interviews. The use of oral corticosteroids characterizing a severe asthma exacerbation^16^ was not described, since the asthma attacks were self‐reported and could not be differentiated into severe and moderate. Information on the prescribed daily dose of ICS was collected from each subject's medical records.

### 2.4 Lung function

Forced expiratory volume in 1 second (FEV_1_) was measured using a Masterscope spirometer (Viasys Healthcare GmbH). Recommendations from the American Thoracic Society were followed.^17^ The percent of predicted values for FEV_1_ and FEV_1_/forced vital capacity (FVC) ratio were calculated on the basis of the Global Lung Function Initiative (GLI) reference values^18^

### 2.5 Exhaled nitric oxide

FeNO measurements were performed in accordance with the American Thoracic Society/European Respiratory Society recommendations,^19^ using a chemiluminescence analyzer (NIOX Flex, Aerocrine AB). The mean value from three exhalations (or two, if they were within 10% of each other) was used for statistical analysis. The analyzer was calibrated every 14 days with certified NO/N_2_ gas of 200 ppb. The percent of predicted FeNO (FeNO%) was calculated using recently developed models, separate for males and females but with children and adults in the same models, adjusted for age and height,^20^ similar to GLI‐adjusted lung function (see above).

### 2.6 Statistics

All statistical analyses were performed using STATA/IC 13.1 (StataCorp LP). If continuous variables had a distribution skewed to the right (e.g., FeNO), a geometric mean with a 95% confidence interval was used for descriptive statistics, and logarithmic transformation was performed before further analyses. Unpaired *t*‐tests (for continuous variables) and chi‐square tests (for categorical variables) were used for the univariate analyses performed for describing the asthmatic subjects at baseline and follow‐up visits. The GLI reference values took both between‐subject and age‐ and height‐related variability into account. We analyzed longitudinal changes (Δ values: follow‐up value minus baseline value) in inflammatory (FeNO, FeNO%, B‐Eos, tIgE, sIgE) and clinical variables (FEV_1_, FEV_1_/FVC, ACT, mAQLQ) between the two visits, based on paired *t*‐test. Longitudinal changes in proportions were analyzed by chi‐square test. Further, we investigated the changes over time of these variables in relation to sIgE concentrations of either Phadiatop or fx5 at baseline, by dividing the asthmatics into three groups: elevated (≥0.35 kU_A_/L), detectable (0.10–0.34 kU_A_/L), and undetectable (<0.10 kU_A_/L) sIgE. Atopy was defined by the presence of either Phadiatop or fx5 above 0.10 kU_A_/L. We performed correlation analyses between the Δ values of different biomarkers, sIgE, and clinical outcomes, using Pearson's test. Zero values of sIgE concentrations were replaced with the value 0.005 before log transformation. Multiple linear regression models with ΔFEV_1_, ΔFEV_1_/FVC, ΔACT, and ΔmAQLQ as dependent variables were constructed for estimating coefficient factors. Independent variables were ∆B‐Eos count, ∆FeNO% (or ∆FeNO), ∆tIgE (or ∆IgE to Phadiatop and fx5), and the analyses were further adjusted for gender, change in age (months), weight, smoking, pet ownership, asthma medication, and ongoing allergen immunotherapy (AIT) at follow‐up. The influence of sex on the association between Δ values of inflammatory biomarkers and clinical outcomes was investigated by interaction analysis. A *p* value < 0.05 was considered statistically significant and *p* < 0.10 indicated a trend.

### 2.7 Ethics

The Uppsala Regional Ethical Review Board approved the study (registration numbers 2009/349 and 2012/420) and all subjects and, when appropriate, their legal guardians gave written informed consent.

## 3 RESULTS

### 3.1 Subject characteristics at baseline and follow‐up

We compared the clinical and inflammatory variables at baseline and follow‐up, a median of 43 months later, in 253 young subjects with asthma. Higher absolute FeNO, lower tIgE, and reduced lung function were noted at follow‐up (Table 1). However, the increase in FeNO disappeared after correcting for individual factors (FeNO%). Furthermore, the subjects had higher ACT and mAQLQ scores, and less frequently reported recent asthma attacks at follow‐up. The asthmatics also used more LTRA and were on higher daily doses of ICS at the follow‐up visit, and the prevalence of any asthma medication use was lower at baseline compared to follow‐up (85.8% and 86.4%, *p* = 0.032). A slightly larger proportion of atopic asthmatics (IgE antibody levels ≥ 0.10 kU_A_/L) was found at baseline compared to follow‐up (87.7% vs. 83.3%), but the difference was not significant (*p* = 0.210). Almost all (99%) of the atopic asthmatic subjects reported allergic symptoms to either furry animals or pollen (data not shown). A larger proportion of the asthmatic subjects who were lost to follow‐up were males and current smokers (Table 2). Mean FeNO and B‐Eos count were calculated by month of examination (except July) with no significant differences between months (data not shown).

**TABLE 1:** Basic characteristics of asthmatic subjects in the MIDAS cohort at baseline and follow‐up after 43 [23–65] months (median [range])

*n* = 253	Baseline	Follow‐up	*p* value
Female (%)	55.4	‐	‐
Age	20.4 ± 7.08	23.9 ± 7.13	n.r.
Weight	63.5 ± 15.9	70.4 ± 14.7	n.r.
Height (cm)	166 ± 12.5	171 ± 9.81	0.223
FeNO (ppb)	15.9 (14.4, 17.5)	18.6 (16.9, 20.4)	0.007
FeNO (%)	111 (107, 115)	116 (112, 120)	0.105
B‐Eos (10^9^/L)	0.179 (0.160, 0.200)	0.174 (0.157, 0.193)	0.116
Phadiatop (kU_A_/L)	5.32 (3.84, 7.37)	4.49 (3.19, 6.33)	0.001
fx5 (kU_A_/L)	0.349 (0.262, 0.467)	0.214 (0.155,0.296)	<0.001
Total IgE (kU/L)	141 (115, 173)	119 (97.7, 145)	0.004
Current smoker (%)	2.39	4.08	0.318
Pet ownership (%)	29%	24%	0.132
ICS (μg daily)	414 (383, 448)	444 (390, 504)	0.048
LTRA (%)	18.8	21.7	<0.001
ACT	20.6 ± 3.26	21.4 ± 3.10	0.001
mAQLQ	5.78 ± 0.967	5.99 ± 0.937	<0.001
FEV_1_ (%)	95.8 ± 13.8	93.6 ± 12.3	<0.001
FVC (%)	100 ± 12.8	99.7 ± 12.1	<0.001
FEV_1_/FVC (%)	94.7 ± 8.59	93.4 ± 9.16	<0.001
Recent asthma attacks (%)	48.0	26.6	<0.001

*Note*: Mean ± SD, Geometric mean (95% CI). Phadiatop: Aeroallergen screening test (nine common aeroallergens), fx5: Food allergy screening test (six common food allergens).

Abbreviations: ACT, Asthma Control Test; B‐Eos, blood eosinophils; FeNO, fraction of exhaled nitric oxide; FEV_1_, forced expiratory volume in 1 second; FVC, forced vital capacity; ICS, inhaled corticosteroid; IgE, immunoglobulin E; LTRA, leukotriene‐receptor antagonist; mAQLQ, mini Asthma‐Related Quality of Life Questionnaire; MIDAS, Minimally Invasive Diagnostic procedures in allergy, Asthma, or food hypersensitivity Study; ppb, parts per billion.

**TABLE 2:** Comparison of baseline characteristics of asthmatic subjects at baseline with asthmatic subjects who dropped‐out in the MIDAS cohort

	Baseline *n* = 253	Drop‐out *n* = 155	*p*
Female (%)	55.4	43.9	0.013
Age	20.4 ± 7.08	20.5 ± 7.04	0.762
Weight (kg)	63.5 ± 15.9	64.8 ± 16.2	0.299
Height (cm)	166 ± 12.5	168 ± 12.3	0.121
FeNO (ppb)	15.9 (14.4, 17.5)	14.8 (13.4,16.5)	0.907
FeNO (%)	111 (107, 115)	118 (106,130)	0.686
B‐Eos (10^9^/L)	0.179 (0.160, 0.200)	0.165 (0.147,0.186)	0.926
Phadiatop (kU_A_/L)	5.32 (3.84, 7.37)	2.29 (1.57,3.35)	0.548
fx5 (kU_A_/L)	0.349 (0.262, 0.467)	0.197 (0.148,0.263)	0.771
Total IgE (kU_A_/L)	141 (115, 173)	105 (83.6,132)	0.913
Current smoker (%)	2.39	7.24	0.018
Pet ownership (%)	29%	31%	0.344
ICS (μg daily)	414 (383, 448)	374 (341,410)	0.086
LTRA (%)	18.8	18.8	0.852
ACT	20.6 ± 3.26	20.2 ± 3.54	0.373
mAQLQ	5.78 ± 0.967	5.75 ± 0.973	0.773
FEV_1_ (%)	95.8 ± 13.8	92.8 ± 13.9	0.756
FVC (%)	100 ± 12.8	98.9 ± 13.5	0.403
FEV_1_/FVC (%)	94.7 ± 8.59	80.5 ± 9.32	0.083
Recent asthma attacks (%)	48.0	44.6	0.496

*Note*: Mean ± SD, Geometric mean (95% CI). Phadiatop: Aeroallergen screening test (nine common aeroallergens), fx5: Food allergy screening test (six common food allergens).

Abbreviations: ACT, Asthma Control Test; B‐Eos, blood eosinophils; FeNO, fraction of exhaled nitric oxide; FEV_1_, forced expiratory volume in 1 second; FVC, forced vital capacity; ICS, inhaled corticosteroid; IgE, immunoglobulin E; LTRA, leukotriene‐receptor antagonist; mAQLQ, mini Asthma‐Related Quality of Life Questionnaire; MIDAS, Minimally Invasive Diagnostic procedures in allergy, Asthma, or food hypersensitivity Study; ppb, parts per billion.

### 3.2 Longitudinal changes in clinical and inflammatory variables stratified by baseline allergen‐specific IgE antibody levels

We investigated the changes in clinical and biomarker variables after stratifying the asthmatics according to baseline sIgE concentrations (Table 3). A significant increase in both FeNO and FeNO% was seen in the elevated sIgE group. A significant decline in lung function was seen in the groups with elevated and detectable sIgE levels (≥0.10 kU_A_/L) but not in the group with undetectable sIgE (<0.10 kU_A_/L) (Table 3). Furthermore, ACT scores improved significantly in the elevated sIgE group, and mAQLQ scores improved in all groups except in the undetectable sIgE group. The level of tIgE declined in the elevated sIgE group, but not the other two groups.

**TABLE 3:** Mean Δ values of clinical variables and inflammatory biomarkers in asthmatic subjects in different groups based on baseline IgE‐antibody concentrations (sIgE – either Phadiatop or fx5)

	IgE ≥ 0.35 kU_A_/L (*n* = 202)		IgE 0.10–0.34 kU_A_/L (*n* = 22)		IgE < 0.10 kU_A_/L (*n* = 29)	
	Δ (mean)	*p*	Δ (mean)	*p*	Δ (mean)	*p*
FeNO (%)	+16.8	0.046	+9.65	0.251	+9.75	0.509
FeNO (ppb)	+7.76	0.011	+1.78	0.105	+4.14	0.669
B‐Eos (10^9^/L)	−0.021	0.067	+0.016	0.506	−0.017	0.915
FEV_1_ (%)	−1.89	0.010	−6.02	0.006	−1.59	0.509
FEV_1_/FVC	−1.11	0.047	−3.43	<0.001	−1.20	0.413
ACT	+0.835	0.005	+1.54	0.156	+0.931	0.202
mAQLQ	+0.145	0.016	+0.590	0.003	+0.254	0.217
Total IgE (kU/L)	−105	0.042	−5.20	0.558	−2.62	0.405
Phadiatop (kU_A_/L)	−7.14	0.001	−0.231	0.933	‐	n.r
fx5 (kU_A_/L)	−1.14	<0.001	−0.035	<0.001	‐	n.r
ICS (μg)	+88	0.152	+160	0.106	+75	0.168
LTRA (%)	+7.51	0.081	+4.92	0.233	+7.66	0.296

*Note*: *p* values from comparison of baseline and follow‐up values (paired *t*‐test).

Abbreviations: ACT, Asthma Control Test; B‐Eos, blood eosinophils; FeNO, fraction of exhaled nitric oxide; FEV_1_, forced expiratory volume in 1 second; FVC, forced vital capacity; ICS, inhaled corticosteroid; IgE, immunoglobulin E; LTRA, leukotriene‐receptor antagonist; mAQLQ, mini Asthma‐Related Quality of Life Questionnaire; n.r, non‐relevant; ppb, parts per billion; sIgE, allergen‐specific IgE antibodies.

### 3.3 Association between longitudinal changes in biomarker and clinical variables

In unadjusted analyses, the longitudinal changes in type‐2 biomarkers and tIgE intercorrelated significantly with each other (Table 4). In contrast, changes in sIgE concentrations did not correlate with changes in type‐2 biomarkers, and only changes in IgE to Phadiatop correlated with tIgE. Furthermore, a significant negative correlation between changes in both B‐Eos and FeNO%, and ΔFEV_1_/FVC was noted.

**TABLE 4:** Correlations between Δ values of clinical and inflammatory biomarkers in asthmatic subjects

*n* = 253	FeNO (%)	FeNO (ppb)	B‐Eos	Total IgE	Phadiatop	fx5
FeNO (%)	‐	0.973**	0.301**	0.162*	0.016	−0.024
FeNO (ppb)	0.973**	‐	0.273**	0.049	0.037	−0.017
B‐Eos	0.301**	0.273**	‐	0.308**	0.018	0.026
Total IgE	0.162*	0.049	0.308**	‐	0.578**	0.157
Phadiatop	0.016	0.037	0.018	0.578**	‐	0.308
fx5	−0.024	−0.017	0.026	0.157	0.308	‐
ACT	−0.029	−0.022	0.088	−0.025	0.014	−0.004
mAQLQ	0.125	0.180	0.031	0.003	0.001	−0.087
FEV_1_ (%)	−0.134*	−0.070	−0.079	−0.032	0.100	0.239
FEV_1_/FVC	−0.056*	−0.167**	−0.175*	0.004	0.110	0.105

Abbreviations: ACT, Asthma Control Test; B‐Eos, blood eosinophils; FeNO, fraction of exhaled nitric oxide; FEV_1_, forced expiratory volume in 1 second; FVC, forced vital capacity; IgE, immunoglobulin E; mAQLQ, mini Asthma‐Related Quality of Life Questionnaire; ppb, parts per billion.

*p < 0.05, **p < 0.001.

**FIGURE 1:**
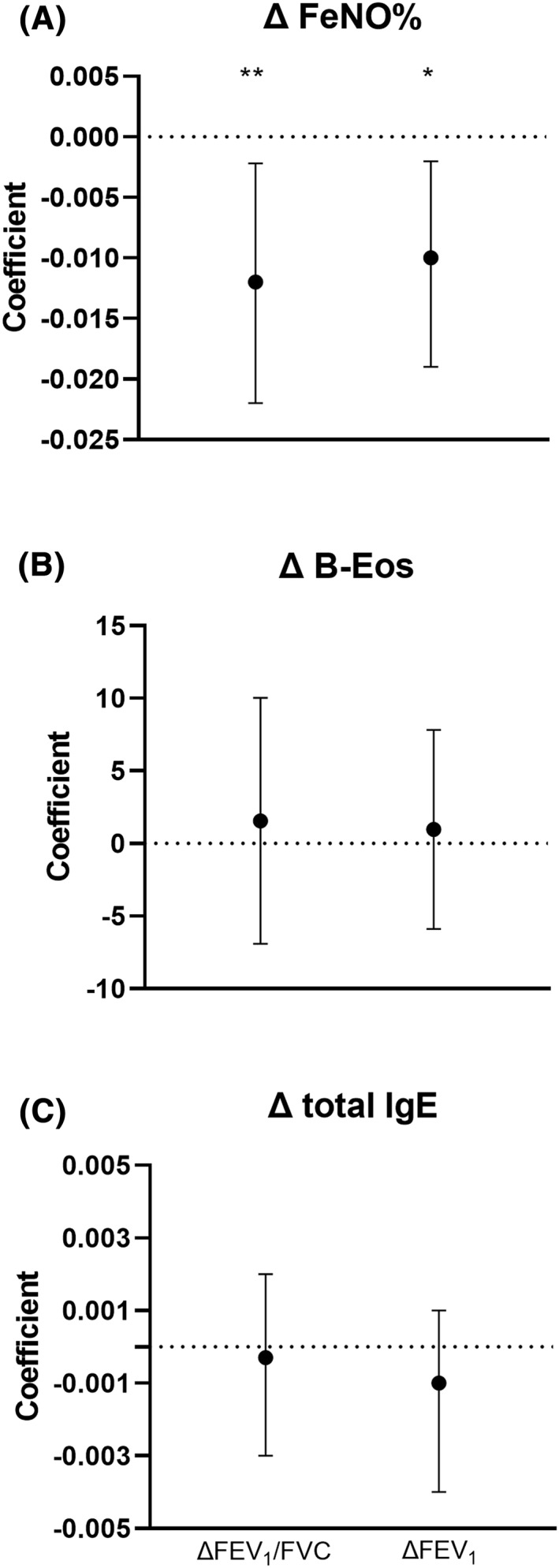
Coefficient factors (beta(95% CI)) for change in inflammatory biomarkers in relation to change in forced expiratory volume in 1 second (FEV1) and FEV1/forced vital capacity in multiple regression analysis performed in asthmatic subjects (IgE ≥ 0.10 kUA/L; *n* = 224). Results were also adjusted for gender, change in age (months), weight, pet ownership, asthma medication, and smoking, and allergen immunotherapy at follow‐up. *p < 0.05, **p < 0.01

We further investigated the relationship between the change in inflammatory variables and IgE antibodies, and lung function using different multiple linear regression models adjusted for confounding factors (sex, change in age, weight, pet ownership, asthma medication, and smoking, as well as AIT at follow‐up). When analyzing the whole asthma population (*n* = 253), an independent relationship was observed between the changes in FeNO%, and FEV_1_/FVC (*p* < 0.01) and FEV_1_ (*p* < 0.05), respectively. Furthermore, these associations were also observed in a model including only asthmatics with sIgE levels ≥0.10 kUA/L (*n* = 224) (Figure 1). Being female was also independently associated with a larger ∆FEV_1_ (*p* = 0.002).

In similar models using absolute FeNO instead of FeNO%, the independent association between change in FeNO and lung function was markedly weakened: FEV_1_/FVC (*p* = 0.071) and FEV_1_ (*p* = 0.094). In similar models again, but with baseline values of the type‐2 biomarkers as independent variables, no significant associations were seen with changes in lung function. No significant associations between changes in type‐2 biomarkers, and changes in mAQLQ and ACT score as dependent variables, were noted in any of the above model variants. For the above multivariate analyses, replacing ΔtIgE with ∆sIgE concentrations to Phadiatop and fx5 did not change the results (data not shown).

### 3.4 Sex difference

We divided the analyzed population into females and males and performed univariate analyses. A significant decline in IgE antibodies to fx5 was observed in both males and females, whereas IgE to Phadiatop and total IgE were significantly decreased only in females. Lung function was the only clinical outcome that was significantly changed over time in both females and males (Table 5). No significant change over time was noted in asthma exacerbations, ACT, and mAQLQ scores when analyzing females and males separately.

**TABLE 5:** Inflammatory biomarkers, sIgE, and clinical outcomes in asthmatic subjects at baseline and follow‐up based on gender

	Females *n* = 140			Males *n* = 113		
	Baseline	Follow‐up	*p*	Baseline	Follow‐up	*p*
FeNO (ppb)	15.5 (13.6,17.7)	17.2 (15.1, 19.5)	0.291	16.4 (14.2, 18.8)	20.6 (18.0, 23.6)	0.322
FeNO%	129 (113, 148)	141 (123, 160)	0.430	130 (113, 149)	150 (131, 170)	0.113
B‐Eos	0,163 (0, 140, 0, 189)	0.162 (0.142, 0.186)	0.538	0.201 (0.170, 0.238)	0.188 (0.160, 0.221)	0.094
Total IgE	124 (93.5, 165)	101 (77.6, 133)	<0.001	168 (126, 223)	145 (107, 195)	0.086
Phadiatop	4.20 (2.64, 6.71)	3.23 (1.98, 5.27)	0.005	7.15 (4.57, 11.2)	6.73 (4.21, 10.8)	0.507
fx5	0.295 (0.207, 0.421)	0.166 (0.111, 0.245)	<0.001	0.423 (0.268, 0.699)	0.294 (0.174, 0.496)	<0.001
FEV_1_ (%)	95.0 (92.7, 97.3)	93.7 (91.3, 95.2)	0.024	94.3 (91.7, 97.0)	91.9 (89.7, 94.2)	0.007
FEV_1_/FVC	94.4 (93.0, 95.6)	93.2 (91.7, 94.7)	0.003	94.2 (92.5, 95.9)	92.8 (90.9, 94.7)	0.024
ACT	20.2 ± 3.10	20.8 ± 3.24	0.422	20.9 ± 3.42	22.9 ± 2.76	0.122
mAQLQ	5.61 ± 0.982	5.80 ± 1.01	0.233	6.01 ± 0.904	6.23 ± 0.795	0.144
Asthma exacerbations	53.5%	33.1%	0.897	41.6%	17.7%	0.066

Abbreviations: ACT, Asthma Control Test; B‐Eos, blood eosinophils; FeNO, fraction of exhaled nitric oxide; FEV_1_, forced expiratory volume in 1 second; FVC, forced vital capacity; IgE, immunoglobulin E; mAQLQ, mini Asthma‐Related Quality of Life Questionnaire; ppb, parts per billion; sIgE, allergen‐specific IgE antibodies.

In sex‐specific analyses, a negative association between ∆FeNO% and ∆FEV_1_ was seen in females (*r* = −0.24, *p* = 0.005) but not males (*r* = −0.003, *p* = 0.97), whereas the association between ∆FeNO% and ∆FEV_1_/FVC became nonsignificant for both sexes. Furthermore, a significant interaction (*p* = 0.02) with sex was found for the relation between changes in FeNO% and changes in FEV_1_. When repeating the multiple linear regression analysis in asthmatics with sIgE levels ≥0.10 kUA/L and stratified according to sex, the independent association between changes in FeNO% and FEV_1_ remained in females (*p* = 0.002), and a similar interaction with gender was seen (*p* = 0.02).

## 4 DISCUSSION

This study describes the longitudinal changes of clinical and inflammatory variables in young asthmatics over a median of 43 months. Despite improved asthma control, lower prevalence of recent asthma attacks at follow‐up, and the fact that the asthmatic subjects used more medication, they were characterized by a small reduction in lung function. This lung function decline was only seen in patients with detectable sIgE levels at baseline. Furthermore, an independent association between an increase in individualized FeNO and lung function decline was shown within this population of relatively well‐controlled asthmatic subjects.

The longitudinal data of type‐2 biomarkers revealed an increase in absolute FeNO. However, it is well‐known that FeNO values increase in parallel with somatic growth and plateaus after puberty.^21^ Accordingly, the increase in FeNO disappeared after adjusting for individual factors including age and height,^20^ when looking at the whole sample of asthmatics. However, the stratification into subgroups based on the sIgE levels at baseline, revealed an increase in individualized FeNO in the group with elevated sIgE levels (≥0.35 kU_A_/L). This could not be shown in a previous study with a shorter follow‐up period of 6 months.^22^ In contrast to FeNO, tIgE declined in and B‐Eos had a tendency to decline in the subgroup with elevated sIgE but not in the groups with lower sIgE levels at baseline. FeNO has previously been suggested to be a reliable marker in the longitudinal assessment of asthma control, particularly in subjects with low doses of ICS, but the ability of FeNO to predict asthma control appears weakened at higher ICS doses.^3^ This finding is in line with our data where, in the context of increased asthma medication at the follow‐up, no correlation was observed between the change in individualized FeNO, and ACT and mAQLQ scores.

Aging may generally be related to a decline in tIgE. In a recent large population‐based study of adults, a reduction in tIgE with increasing age was observed regardless of the age at baseline,^23^ which appears to be consistent with our data. One possible explanation for this could be the age‐associated alterations of the immune system leading to a depression of T‐cell function.^24^ Although both T cells and B cells are involved in the onset of atopy and the regulation of IgE formation, it is unknown to what extent this regulation is T cell dependent.^25^, ^26^ In our study, the stratification according to sIgE levels at baseline showed that higher sIgE levels were more likely to predict a decline in tIgE over time. A reduction in the prevalence of IgE sensitization and possible allergen avoidance might also have influenced tIgE as the cohort aged. Evidence supporting our data comes from a large cross‐sectional study where tIgE was highest among young children (aged 6–9 years), and decreased progressively as a function of age.^27^ Our findings though contrast with Patelis et al., who showed that tIgE increased in adults, aged 20–45 years at baseline, in a large cohort followed up after 9 years.^28^ However, we have to take into consideration that our cohort involved younger subjects, including children and adolescents.

Another important observation derived from the stratification into different sIgE groups was that a significant reduction in lung function over time was seen in the elevated and detectable sIgE groups but was absent in the group with undetectable sIgE. This finding is supported by our recent study, highlighting the presence of clinically significant type‐2 inflammation in patients with low but detectable sIgE levels (0.10–0.34 kU_A_/L) but not in patients with undetectable sIgE.^9^ The cut‐off of 0.10 kU_A_/L for ruling out IgE sensitization used in this study contrasts with clinical routine where a higher cut‐off level of 0.35 kU_A_/L or a wheal diameter for skin prick test greater than 3 mm is commonly used for defining atopy. However, a lower cut‐off of >0 mm for skin prick tests seems to increase the sensitivity for identifying IgE sensitization, applicable though primarily in epidemiological studies.^29^

According to earlier longitudinal studies, a high level of tIgE measured at baseline has been related to reduced lung function, but attempts have failed to link the deterioration of lung function over time to baseline tIgE, implying that other factors drive lung function decline in asthmatic subjects.^7^, ^30^ In agreement with these studies, we could not see any independent associations between changes in lung function and changes in tIgE. The relationship between longitudinal changes in lung function and B‐Eos count, a biomarker of systemic type‐2 inflammation, has scarcely been studied, but it was recently suggested that there is only a weak relationship between B‐Eos and decline in lung function.^31^

In contrast, we were able to show an independent association between the change in individualized FeNO and lung function decline, when introducing FeNO%, B‐Eos count and tIgE in the same multivariate models. Previous studies have shown a correlation between elevated baseline FeNO and accelerated lung function decline over 5 years, whereas the correlation with baseline sputum eosinophil count was less consistent.^32^, ^33^ However, the independence of these correlations was not tested in those studies, and we could not find any independent associations between the baseline level of any type‐2 biomarker and lung function decline in our study.

With regard to lung function decline over time, we have to take into account the normal aging process in the lungs including the loss of elastic recoil, alterations in gas exchange and a lung growth rate which decelerates by the age of 20–25 years.^34^, ^35^ However, lung function variables were adjusted according to GLI, with seamless reference equations throughout the entire age range.^18^ Thus, the changes in lung function reported here should be considered accelerated decline on top of normal physiological changes.

We hypothesize that the higher asthma medication use noted in our asthmatic subjects at the 43‐month follow‐up visit, could be a study effect in the form of improved medication adherence. In addition, the transition from childhood to adulthood asthma as the cohort aged, including a faster decline in lung function and appearance of new trigger factors such as pharmaceutical and occupational agents,^36^, ^37^ might also contribute to explaining the higher use of asthma medication to attain adequate asthma control. Moreover, and according to guidelines, adults require higher ICS daily doses than children in maintenance treatment. It has previously been reported that the use of ICS may reduce the lung function decline in males, but not females, with moderate to severe asthma.^38^ In agreement with this, the association between lung function decline and individualized FeNO was observed only in female asthmatics. Our findings that males had higher sIgE concentrations to aeroallergens, and that these antibody levels declined in females but not males, is consistent with the general view that atopy is a more important risk factor for asthma in males compared to females among young subjects.^39^ Interestingly, individualized FeNO increased while sIgE concentrations decreased during the study. Many studies have shown a correlation between the degree of IgE sensitization and FeNO.^40^ Our data indicate that clinically relevant mucosal type‐2 inflammation can worsen even though sIgE concentrations decrease. However, the proportion of asthmatics with detectable sIgE levels was not significantly changed during the study.

We chose to include also IgE to food allergens in the present study. This was based on previous findings that food allergens can cause both acute and late‐phase airway reactions via the inhalational route in food‐allergic individuals,^41^ and that food IgE sensitization associates with both FeNO and B‐Eos count, independently of aeroallergen sensitization.^10^

A strength of our study was the relatively long follow‐up period, a median of 43 months, and our study benefits from the mixed recruitment of young asthmatics from both primary and specialist care. Another strength of this study was the application of individualized FeNO based on a model similar to GLI‐adjusted lung function. Furthermore, this is, to our knowledge, the first longitudinal study assessing sIgE levels against food and aeroallergens, total IgE and type‐2 biomarkers, and the association with clinical changes, in patients with asthma. A limitation of the study could be the follow‐up rate of 62%, which may have resulted in selection bias. However, those lost to follow‐up differed from the included subjects only by having a larger proportion of males and current smokers, findings in line with similar studies.^42^

In conclusion, in this cohort of young asthmatics followed over a median of 43 months, IgE was reduced, but B‐Eos and FeNO remained unchanged during the observation period. An accelerated lung function decline was seen in subjects with detectable sIgE levels (≥0.10 kU_Α_/L) but not in subjects with undetectable sIgE. An independent association between increase in individualized FeNO and lung function decline was seen in female asthmatics. Our findings suggest that exhaled NO signals for inflammatory mechanisms closely related to accelerated lung function decline independent of IgE levels and B‐Eos count.

## CONFLICT OF INTEREST

Magnus Borres is an employee of Thermo Fisher Scientific, and Kjell Alving has received research material from the same company and from Hemocue. None of the other authors declare conflict of interest.

## References

[1] Silkoff PE , Laviolette M , Singh D , et al. Longitudinal stability of asthma characteristics and biomarkers from the Airways Disease Endotyping for Personalized Therapeutics (ADEPT) study. Respir Res. 2016;17:43. 27107814 10.1186/s12931-016-0360-5 PMC4842260

[2] van der Valk RJ , Baraldi E , Stern G , Frey U , de Jongste JC . Daily exhaled nitric oxide measurements and asthma exacerbations in children. Allergy. 2012;67(2):265‐271. 21999328 10.1111/j.1398-9995.2011.02734.x

[3] Michils A , Baldassarre S , Van Muylem A . Exhaled nitric oxide and asthma control: a longitudinal study in unselected patients. Eur Respir J. 2008;31(3):539‐546. 18057062 10.1183/09031936.00020407

[4] Price DB , Rigazio A , Campbell JD , et al. Blood eosinophil count and prospective annual asthma disease burden: a UK cohort study. Lancet Respir Med. 2015;3(11):849‐858. 26493938 10.1016/S2213-2600(15)00367-7

[5] Zeiger RS , Schatz M , Li Q , et al. High blood eosinophil count is a risk factor for future asthma exacerbations in adult persistent asthma. J Allergy Clin Immunol Pract. 2014;2(6):741‐750. 25439366 10.1016/j.jaip.2014.06.005

[6] Porsbjerg C , Lange P , Ulrik CS . Lung function impairment increases with age of diagnosis in adult onset asthma. Respir Med. 2015;109(7):821‐827. 25962648 10.1016/j.rmed.2015.04.012

[7] Shadick NA , Sparrow D , O'Connor GT , DeMolles D , Weiss ST . Relationship of serum IgE concentration to level and rate of decline of pulmonary function: the Normative Aging Study. Thorax. 1996;51(8):787‐792. 8795665 10.1136/thx.51.8.787 PMC472538

[8] Turner S , Fielding S , Mullane D , et al. A longitudinal study of lung function from 1 month to 18 years of age. Thorax. 2014;69(11):1015‐1020. 24891326 10.1136/thoraxjnl-2013-204931

[9] Tsolakis N , Malinovschi A , Nordvall L , Janson C , Borres MP , Alving K . The absence of serum IgE antibodies indicates non‐type 2 disease in young asthmatics. Clin Exp Allergy. 2018;48(6):722‐730. 29377450 10.1111/cea.13103

[10] Patelis A , Janson C , Borres MP , Nordvall L , Alving K , Malinovschi A . Aeroallergen and food IgE sensitization and local and systemic inflammation in asthma. Allergy. 2014;69(3):380‐387. 24397423 10.1111/all.12345

[11] Heijkenskjold‐Rentzhog C , Nordvall L , Janson C , Borres MP , Alving K , Malinovschi A . Alveolar and exhaled NO in relation to asthma characteristics – effects of correction for axial diffusion. Allergy. 2014;69(8):1102‐1111. 24894594 10.1111/all.12430

[12] Vidal C , Gude F , Boquete O , et al. Evaluation of the Phadiatop test in the diagnosis of allergic sensitization in a general adult population. J Investig Allergol Clin Immunol. 2005;15(2):124‐130. 16047713

[13] Siroux V , Boudier A , Anto JM , et al. Quality‐of‐life and asthma‐severity in general population asthmatics: results of the ECRHS II study. Allergy. 2008;63(5):547‐554. 18394129 10.1111/j.1398-9995.2008.01638.x

[14] Nathan RA , Sorkness CA , Kosinski M , et al. Development of the Asthma Control Test: a survey for assessing asthma control. J Allergy Clin Immunol. 2004;113(1):59‐65. 14713908 10.1016/j.jaci.2003.09.008

[15] Juniper EF , Guyatt GH , Cox FM , Ferrie PJ , King DR . Development and validation of the Mini Asthma Quality of Life Questionnaire. Eur Respir J. 1999;14(1):32‐38. 10489826 10.1034/j.1399-3003.1999.14a08.x

[16] Reddel HK , Taylor DR , Bateman ED , et al. An official American Thoracic Society/European Respiratory Society statement: asthma control and exacerbations: standardizing endpoints for clinical asthma trials and clinical practice. Am J Respir Crit Care Med. 2009;180(1):59‐99. 19535666 10.1164/rccm.200801-060ST

[17] Standardization of Spirometry, 1994 Update. American Thoracic Society 10.1164/ajrccm.152.3.7663792 7663792

[18] Quanjer PH , Stanojevic S , Cole TJ , et al. Multi‐ethnic reference values for spirometry for the 3–95‐yr age range: the global lung function 2012 equations. Eur Respir J. 2012;40(6):1324‐1343. 22743675 10.1183/09031936.00080312 PMC3786581

[19] American Thoracic, S. and S. European Respiratory . ATS/ERS recommendations for standardized procedures for the online and offline measurement of exhaled lower respiratory nitric oxide and nasal nitric oxide. Am J Respir Crit Care Med. 2005;171(8):912‐930. 15817806 10.1164/rccm.200406-710ST

[20] Jacinto T , Amaral R , Malinovschi A , Janson C , Fonseca J , Alving K . Exhaled NO reference limits in a large population‐based sample using the lambda‐mu‐sigma method. J Appl Physiol. 2018;125(5):1620‐1626. 30161011 10.1152/japplphysiol.00093.2018

[21] Jacinto T , Malinovschi A , Janson C , Fonseca J , Alving K . Evolution of exhaled nitric oxide levels throughout development and aging of healthy humans. J Breath Res. 2015;9(3):036005. 25993061 10.1088/1752-7155/9/3/036005

[22] Elmasri M , Romero KM , Gilman RH , et al. Longitudinal assessment of high versus low levels of fractional exhaled nitric oxide among children with asthma and atopy. Lung. 2014;192(2):305‐312. 24414739 10.1007/s00408-013-9551-8 PMC5526602

[23] Amaral AFS , Newson RB , Abramson MJ , et al. Changes in IgE sensitization and total IgE levels over 20 years of follow‐up. J Allergy Clin Immunol. 2016;137(6):1788‐1795. 26586040 10.1016/j.jaci.2015.09.037 PMC4889785

[24] Scichilone N , Callari A , Augugliaro G , Marchese M , Togias A , Bellia V . The impact of age on prevalence of positive skin prick tests and specific IgE tests. Respir Med. 2011;105(5):651‐658. 21220195 10.1016/j.rmed.2010.12.014

[25] Jackola DR , Pierson‐Mullany LK , Daniels LR , Corazalla E , Rosenberg A , Blumenthal MN . Robustness into advanced age of atopy‐specific mechanisms in atopy‐prone families. J Gerontol A Biol Sci Med Sci. 2003;58(2):99‐107. 12586846 10.1093/gerona/58.2.b99

[26] Mediaty A , Neuber K . Total and specific serum IgE decreases with age in patients with allergic rhinitis, asthma and insect allergy but not in patients with atopic dermatitis. Immun Ageing. 2005;2(1):9. 15927080 10.1186/1742-4933-2-9 PMC1156931

[27] Salo PM , Calatroni A , Gergen PJ , et al. Allergy‐related outcomes in relation to serum IgE: results from the National Health and Nutrition Examination Survey 2005–2006. J Allergy Clin Immunol. 2011;127(5):1226‐1235. 21320720 10.1016/j.jaci.2010.12.1106 PMC3108140

[28] Patelis A , Gunnbjörnsdottir M , Borres MP , et al. Natural history of perceived food hypersensitivity and IgE sensitisation to food allergens in a cohort of adults. PLoS One. 2014;9(1):e85333. 24427301 10.1371/journal.pone.0085333 PMC3888405

[29] Bousquet PJ , Chatzi L , Jarvis D , Burney P . Assessing skin prick tests reliability in ECRHS‐I. Allergy. 2008;63(3):341‐346. 18070229 10.1111/j.1398-9995.2007.01581.x

[30] Ulrik CS . Outcome of asthma: longitudinal changes in lung function. Eur Respir J. 1999;13(4):904‐918. 10362061 10.1034/j.1399-3003.1999.13d35.x

[31] Brightling CE , George L . Is the eosinophil a leading villain in lung function decline? Chest. 2015;148(4):844‐846. 26437813 10.1378/chest.15-0915

[32] van Veen IH , Ten Brinke A , Sterk PJ , et al. Exhaled nitric oxide predicts lung function decline in difficult‐to‐treat asthma. Eur Respir J. 2008;32(2):344‐349. 18508818 10.1183/09031936.00135907

[33] Contoli M , Baraldo S , Marku B , et al. Fixed airflow obstruction due to asthma or chronic obstructive pulmonary disease: 5‐year follow‐up. J Allergy Clin Immunol. 2010;125(4):830‐837. 20227753 10.1016/j.jaci.2010.01.003

[34] Sharma G , Goodwin J . Effect of aging on respiratory system physiology and immunology. Clin Interv Aging. 2006;1(3):253‐260. 18046878 10.2147/ciia.2006.1.3.253 PMC2695176

[35] Skloot GS . The effects of aging on lung structure and function. Clin Geriatr Med. 2017;33(4):447‐457. 28991643 10.1016/j.cger.2017.06.001

[36] Trupin L , Balmes JR , Chen H , et al. An integrated model of environmental factors in adult asthma lung function and disease severity: a cross‐sectional study. Environ Health. 2010;9:24. 20487557 10.1186/1476-069X-9-24 PMC2887801

[37] Burdon J . Adult‐onset asthma. Aust Fam Physician. 2015;44(8):554‐557. 26510141

[38] Dijkstra A , Vonk JM , Jongepier H , et al. Lung function decline in asthma: association with inhaled corticosteroids, smoking and sex. Thorax. 2006;61(2):105‐110. 16308336 10.1136/thx.2004.039271 PMC2104585

[39] Kalm‐Stephens P , Nordvall L , Janson C , Neumann A , Malinovschi M , Alving K . Different baseline characteristics are associated with incident wheeze in female and male adolescents. Acta Paediatr. 2020;109(11):2324‐2331. 32187749 10.1111/apa.15263

[40] Sacco O , Sale R , Silvestri M , et al. Total and allergen‐specific IgE levels in serum reflect blood eosinophilia and fractional exhaled nitric oxide concentrations but not pulmonary functions in allergic asthmatic children sensitized to house dust mites. Pediatr Allergy Immunol. 2003;14(6):475‐481. 14675476 10.1046/j.0905-6157.2003.00092.x

[41] Roberts G , Golder N , Lack G . Bronchial challenges with aerosolized food in asthmatic, food‐allergic children. Allergy. 2002;57(8):713‐717. 12121190 10.1034/j.1398-9995.2002.03366.x

[42] Ronmark EP , Ekerljung L , Lötvall J , Torén K , Rönmark E , Lundbäck B . Large scale questionnaire survey on respiratory health in Sweden: effects of late‐ and non‐response. Respir Med. 2009;103(12):1807‐1815. 19695859 10.1016/j.rmed.2009.07.014

